# Supplementary material for: Self-regulated reversal deformation and locomotion of structurally homogenous hydrogels subjected to constant light illumination
Source: Nat Commun. 2024 Feb 24;15:1694. doi: 10.1038/s41467-024-46100-6 (PMC10894256; doi:10.1038/s41467-024-46100-6)
Supplement: Supplementary file 1 — Supplementary Information [file 41467_2024_46100_MOESM1_ESM.pdf]

Supplementary Information

Self-regulated reversal deformation and locomotion of structurally homogenous hydrogels subjected to constant light illumination

Kexin Guo[1,2,3#], Xuehan Yang[1#], Chao Zhou[2,3], Chuang Li[1*]

[1]*Key Laboratory of Precision and Intelligent Chemistry, Department of Polymer Science and Engineering, University of Science and Technology of China, Hefei 230026, China.*

[2]*CAS Key Laboratory of Nano-Bio Interface, Division of Nanobiomedicine and i-Lab, Suzhou Institute of Nano-Tech and Nano-Bionics, Chinese Academy of Sciences, Suzhou 215123, China.*

[3]*School of Nano-Tech and Nano-Bionics, University of Science and Technology of China, Hefei 230026, China.*

[*]*Correspondence: lichuang21@ustc.edu.cn*

[#]*These authors contributed equally to this work.*

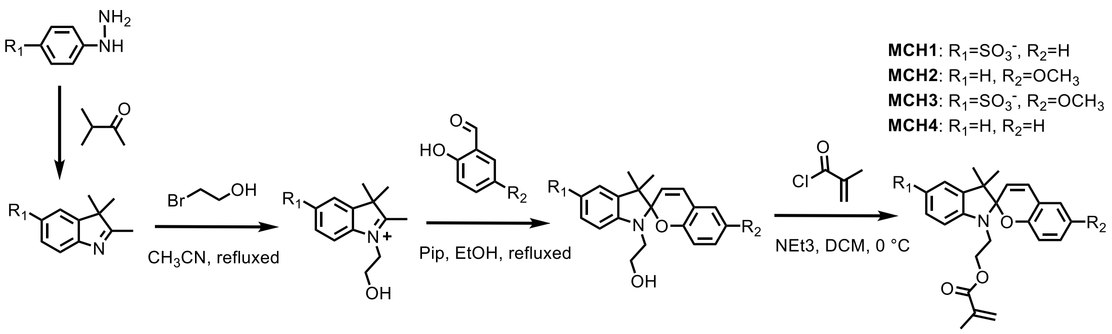

Supplementary Fig. 1. General synthetic route of spiropyran MCH1-MCH4.

Supplementary Table 1. Formulations for preparation of MCH(1+2) hydrogels with variable mixing ratios.

MCH1:MCH2	NIPAAm/g	MBAAm/mg	MCH1/mg	MCH2/mg
2.0:0.0	0.1	6.8	8.74	0.00
1.5:0.5	0.1	6.8	6.55	1.79
1.2:0.8	0.1	6.8	5.24	2.87
1.0:1.0	0.1	6.8	4.37	3.58
0.8:1.2	0.1	6.8	3.49	4.30
0.5:1.5	0.1	6.8	2.18	5.38
0.0:2.0	0.1	6.8	0.00	7.17

Supplementary Table 2. Formulations for the preparation of MCH(1+2) hydrogels containing variable grafting densities (MCH1:MCH2 = 1:1).

Grafting density	NIPAAm/g	MBAAm/mg	MCH1/mg	MCH2/mg
2.5%	0.1	6.8	5.46	4.48
2.0%	0.1	6.8	4.37	3.58
1.5%	0.1	6.8	3.28	2.69
1.0%	0.1	6.8	2.18	1.79

Supplementary Table 3. Formulations for preparation of MCH(3+2) hydrogels with variable mixing ratios.

MCH3:MCH2	NIPAAm/g	MBAAm/mg	MCH3/mg	MCH2/mg
2.0:0.0	0.1	6.8	9.27	0.00
1.5:0.5	0.1	6.8	6.95	1.79
1.2:0.8	0.1	6.8	5.56	2.87
1.0:1.0	0.1	6.8	4.63	3.58
0.8:1.2	0.1	6.8	3.71	4.30
0.5:1.5	0.1	6.8	2.32	5.38
0.0:2.0	0.1	6.8	0.00	7.17

Supplementary Table 4. Formulations for the preparation of MC(3+2) hydrogels containing variable grafting densities (MCH3:MCH2 = 1:1).

Grafting density	NIPAAm/g	MBAAm/mg	MCH3/mg	MCH2/mg
2.5%	0.1	6.8	5.79	4.48
2.0%	0.1	6.8	4.63	3.58
1.5%	0.1	6.8	3.48	2.69
1.0%	0.1	6.8	2.32	1.79

Supplementary Table 5. Formulations for preparation of MCH(3+4) hydrogels with variable mixing ratios.

MCH3:MCH4	NIPAAm/g	MBAAm/mg	MCH3/mg	MCH4/mg
2.0:0.0	0.1	6.8	9.27	0.00
1.5:0.5	0.1	6.8	6.95	1.66
1.2:0.8	0.1	6.8	5.56	2.66
1.0:1.0	0.1	6.8	4.63	3.32
0.8:1.2	0.1	6.8	3.71	3.99
0.5:1.5	0.1	6.8	2.32	4.98
0.0:2.0	0.1	6.8	0.00	6.64

Supplementary Table 6. Formulations for preparation of MCH(1+4) hydrogels with variable mixing ratios.

MCH1:MCH4	NIPAAm/g	MBAAm/mg	MCH1/mg	MCH4/mg
2.0:0.0	0.1	6.8	8.74	0.00
1.5:0.5	0.1	6.8	6.55	1.66
1.2:0.8	0.1	6.8	5.24	2.66
1.0:1.0	0.1	6.8	4.37	3.32
0.8:1.2	0.1	6.8	3.49	3.99
0.5:1.5	0.1	6.8	2.18	4.98
0.0:2.0	0.1	6.8	0.00	6.64

1'-(2-(methacryloyloxy) ethyl)-3',3'-dimethylspiro[chromene-2,2'-indoline]-5'-sulfonate **(MCH1)**

^{1}H-NMR (400 MHz, DMSO-d_6) δ(ppm): 7.39 (dd, J = 8.0, 1.7 Hz, 1H), 7.30 (d, J = 1.7 Hz, 1H), 7.17 (dd, J = 7.5, 1.7 Hz, 1H), 7.09 (td, J = 7.7, 1.7 Hz, 1H), 7.00 (d, J = 10.2 Hz, 1H), 6.84 (td, J = 7.4, 1.1 Hz, 1H), 6.68 (d, J = 8.0 Hz, 1H), 6.61 (d, J = 8.1 Hz, 1H), 6.01 (dd, J = 1.8, 1.0 Hz, 1H), 5.76 (d, J = 10.2 Hz, 1H), 5.67 (p, J = 1.7 Hz, 1H), 4.29 (ddd, J = 11.8, 7.1, 5.0 Hz, 1H), 4.20 (dt, J = 11.1, 5.4 Hz, 1H), 3.53-3.38 (m, 2H), 1.85 (d, J = 1.2 Hz, 3H), 1.20 (s, 3H), 1.06 (s, 3H).

^{13}C NMR (400 MHz, DMSO-d6) δ(ppm): 166.89, 153.98, 147.56, 140.05, 136.15, 135.53, 130.33, 129.87, 127.48, 126.47, 125.64, 120.84, 119.92, 119.53, 118.76, 114.95, 105.35, 104.85, 63.03, 52.12, 42.48, 25.84, 20.02, 18.50.

MS-ESI (m/z): [M] calc. for $C_{24}H_{24}NO_6S^-$ 454.52; $[M+2H]^{2+}$ found 456.20.

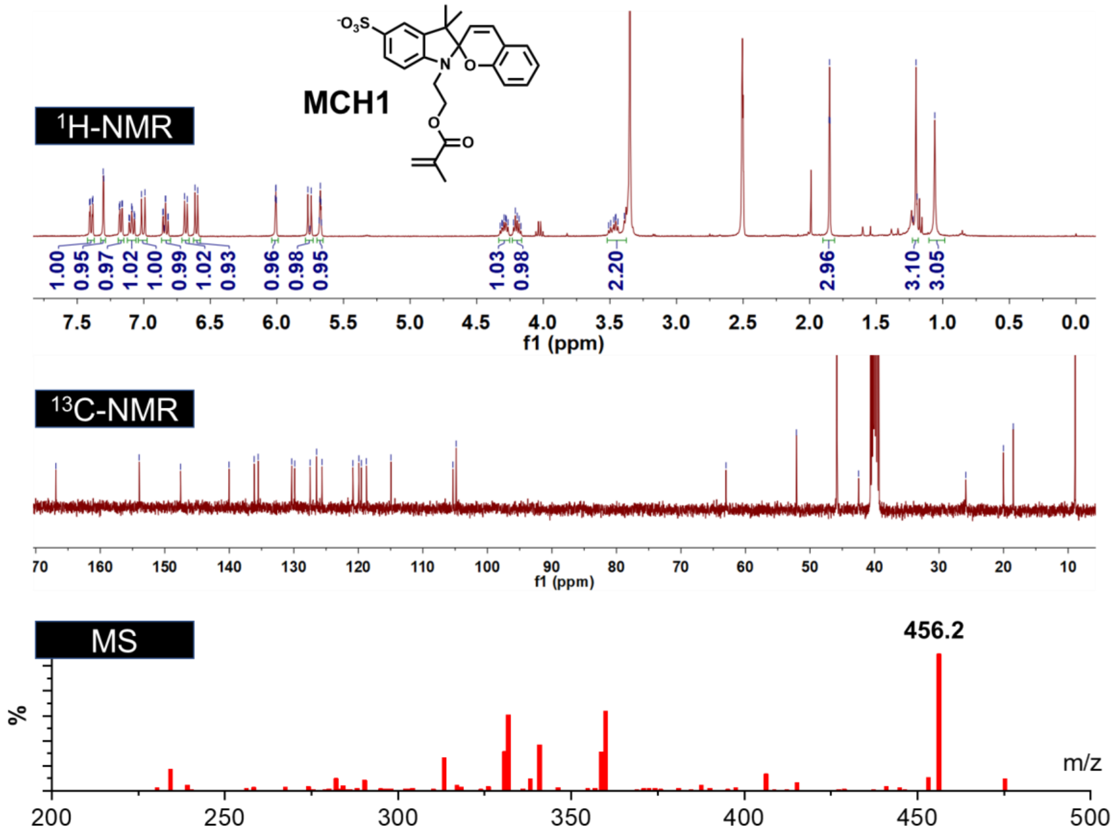

Supplementary Fig. 2. Characterization of MCH1. ^{1}H-NMR (top), ^{13}C NMR (middle) and MS spectra (bottom) of MCH1.

2-(6-Methoxy-3',3'-dimethylspiro[chromene-2,2'-indolin]-1'-yl) ethyl methacrylate **(MCH2)**

^{1}H-NMR (400 MHz, $CDCl_3$) δ(ppm): 7.17 (tt, J = 7.6, 1.3 Hz, 1H), 7.06 (dd, J = 7.3, 1.3 Hz, 1H), 6.84 (td, J = 7.4, 1.0 Hz, 1H), 6.78 (d, J = 10.1 Hz, 1H), 6.69–6.57 (m, 4H), 6.08 (dt, J = 1.7, 1.0 Hz, 1H), 5.71 (d, J = 10.2 Hz, 1H), 5.55 (p, J = 1.6 Hz, 1H), 4.29 (t, J = 6.4 Hz, 2H), 3.75 (s, 3H), 3.59 (dt, J = 15.1, 6.7 Hz, 1H), 3.39 (dt, J = 15.1, 6.1 Hz, 1H), 1.92 (t, J = 1.3 Hz, 3H), 1.29 (s, 3H), 1.13 (s, 3H).

^{13}C-NMR (400 MHz, $CDCl_3$) δ(ppm): 167.32, 153.23, 148.18, 147.23, 136.41, 136.18, 129.37, 127.55, 125.74, 121.72, 120.49, 119.16, 118.76, 115.60, 115.33, 111.58, 106.42, 104.17, 63.04, 55.79, 52.14, 42.42, 25.84, 20.12, 18.40.

MS-ESI (m/z): [M] calc. for $C_{25}H_{27}NO_4$ 405.49; $[M+H]^+$ found 406.20.

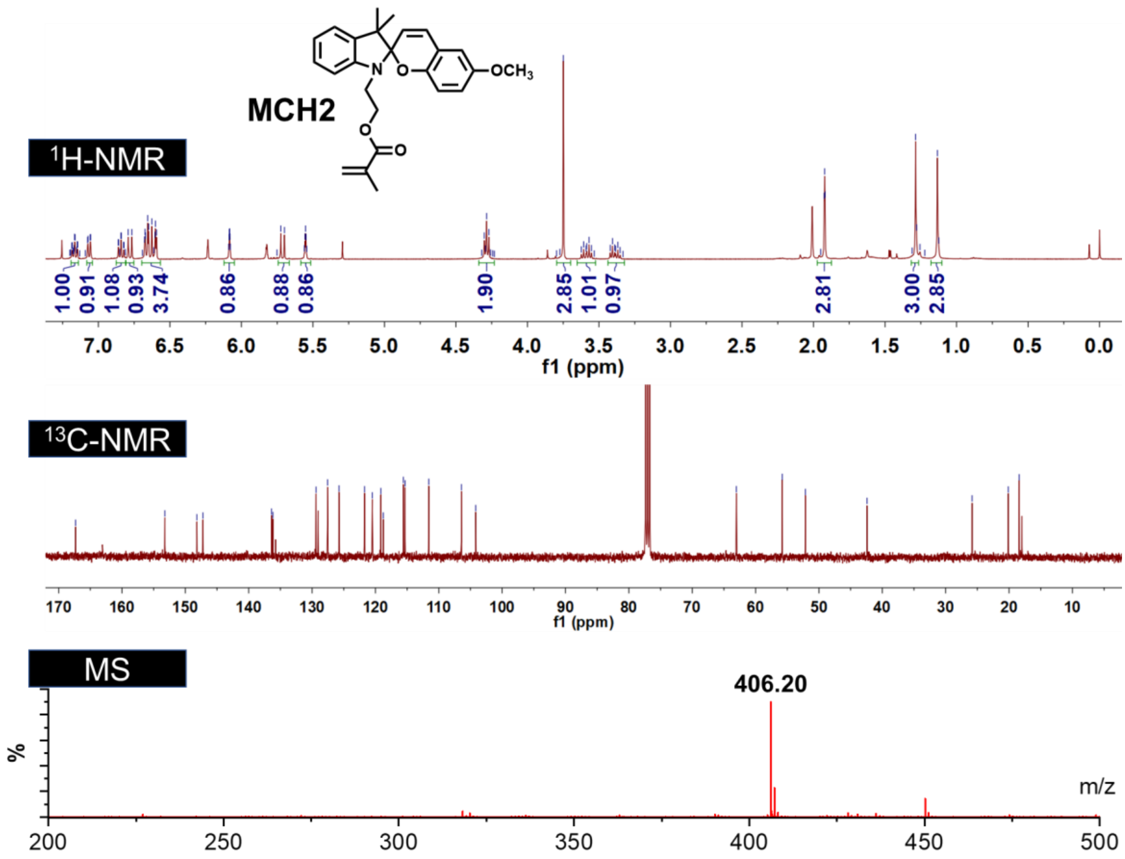

Supplementary Fig. 3. **Characterization of MCH12.** ^{1}H-NMR (top), ^{13}C NMR (middle) and MS spectra (bottom) of MCH2.

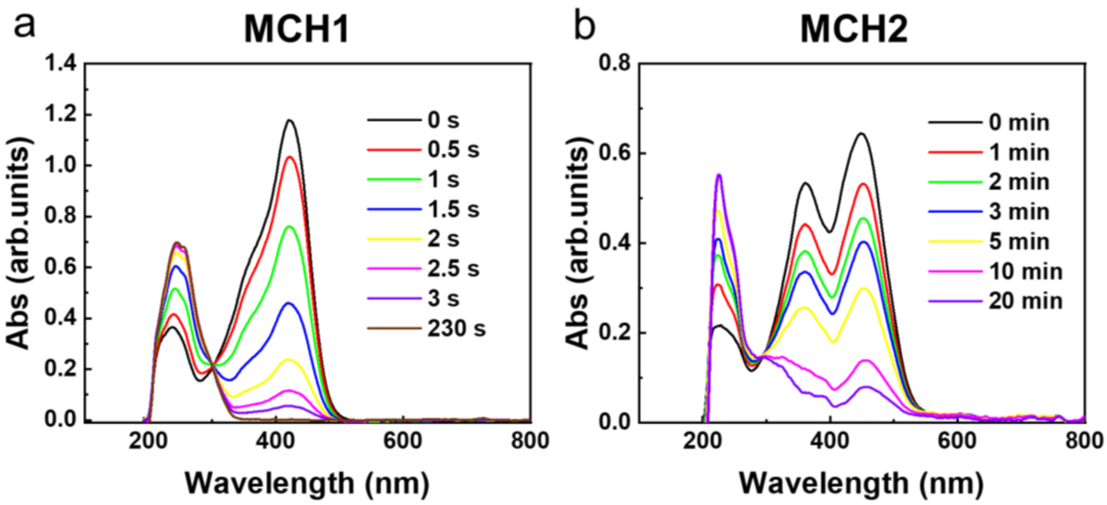

Supplementary Fig. 4. **UV–Vis absorption spectra.** (a) MCH1 and (b) MCH2 were meaarued in a solvent mixture of methanol/water (4:1, v/v) upon irradiation (450 nm, 154.6 mW/cm^2) for various durations.

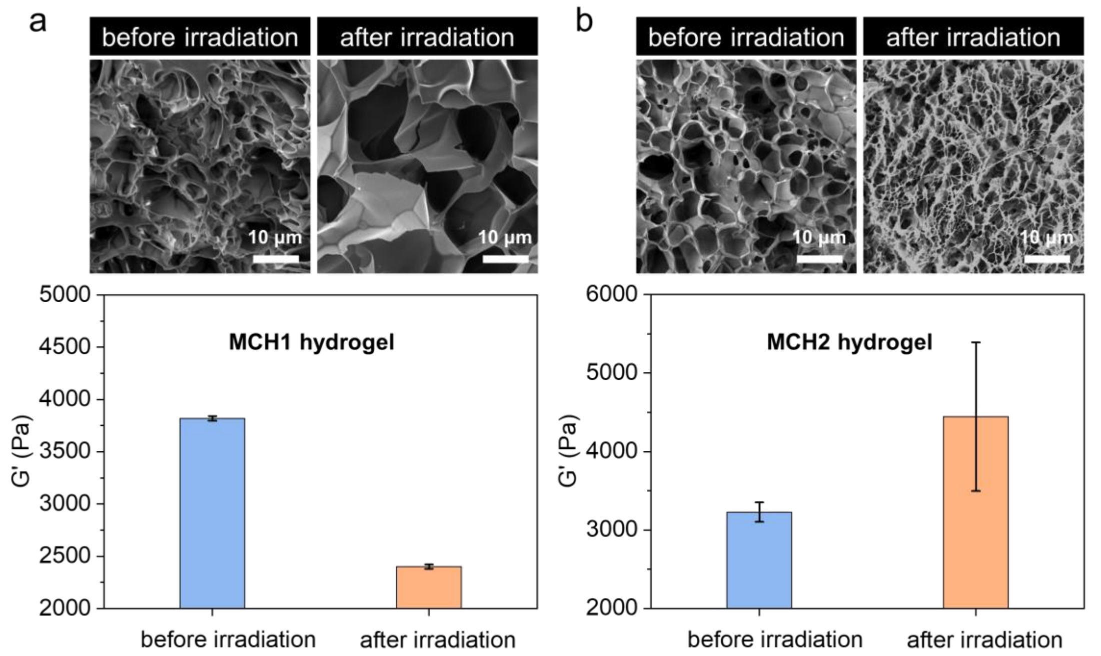

Supplementary Fig. 5. **Mechanical and morphological change of single-component hydrogels upon irradiation.** (a) Rheological measurements of single-component MCH1 hydrogels before (blue) and after irradiation (450 nm, 8.99 mW/cm²) for 90 min (orange). The top images are corresponding SEM images showing the changes in microporosity. (b) Rheological measurements of single-component MCH2 hydrogels before (blue) and after irradiation (450 nm, 8.99 mW/cm²) for 90 min (orange). The top images are corresponding SEM images showing the changes in microporosity. Error bars represent standard deviations of data collected from three separate samples.

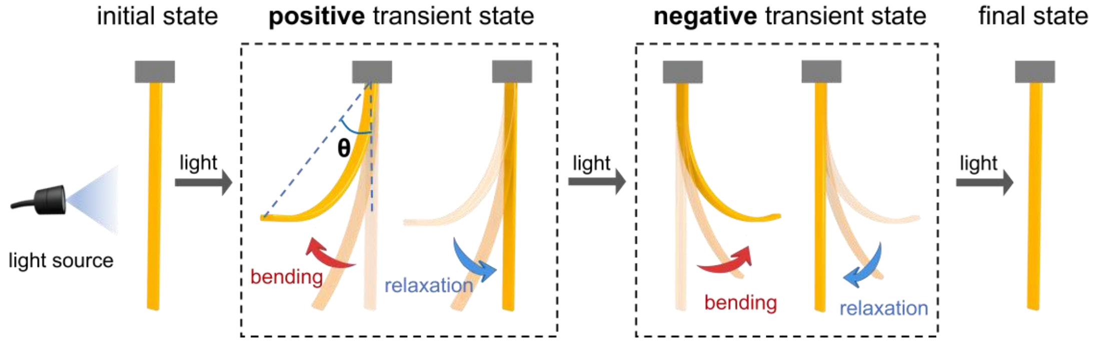

Supplementary Fig. 6. Schematic representation of transient bidirectional positive and negative bending deformation of a ribbon-shaped MCH(1+2) hydrogel upon irradiation from the left. The bending angle is defined as θ.

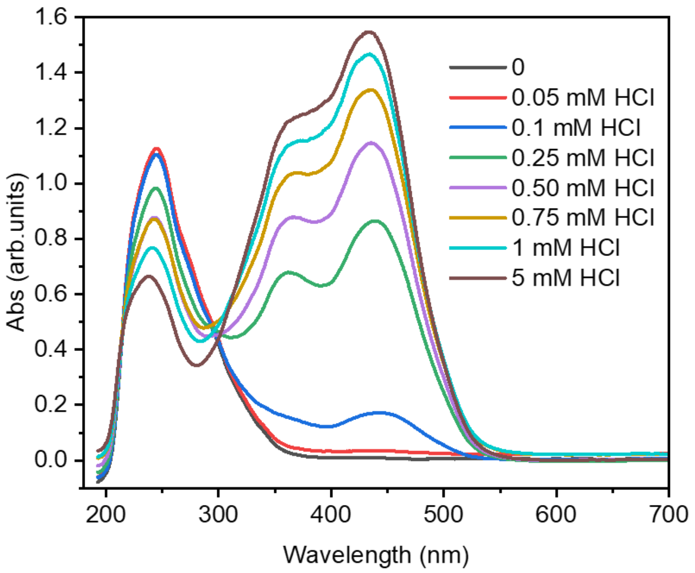

Supplementary Fig. 7. UV–Vis absorption spectra of mixed MCH1 and MCH2 with a fixed mixing molar ratio of 1:1 (total concentration of 0.1 mM) in a mixture in a solvent of methanol/water (4:1, v/v) containing variable concentrations of HCl after equilibrium in the dark.

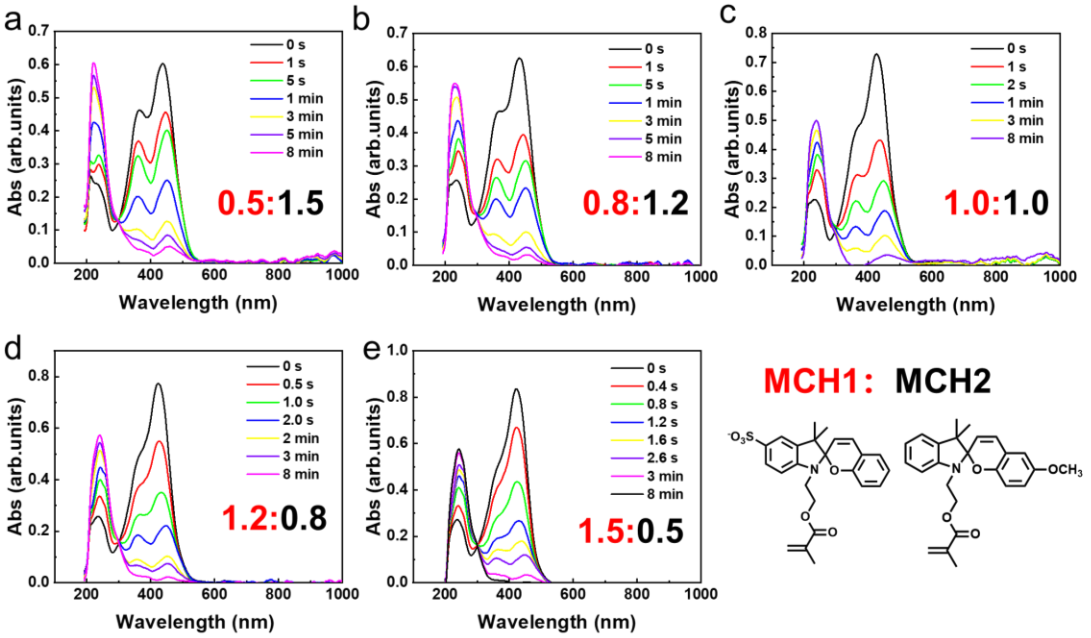

Supplementary Fig. 8. **UV–Vis absorption spectra of mixed MCH1 and MCH2 with variable mixing molar ratios, (a) 0.5:1.5, (b) 0.8:1.2, (c) 1.0:1.0, (d) 1.2:0.8 and (e) 1.5:0.5.** The mearuements were perfomred in a solvent mixture of methanol/water (4:1, v/v) upon irradiation (450 nm, 154.6 mW/cm^2) for different durations.

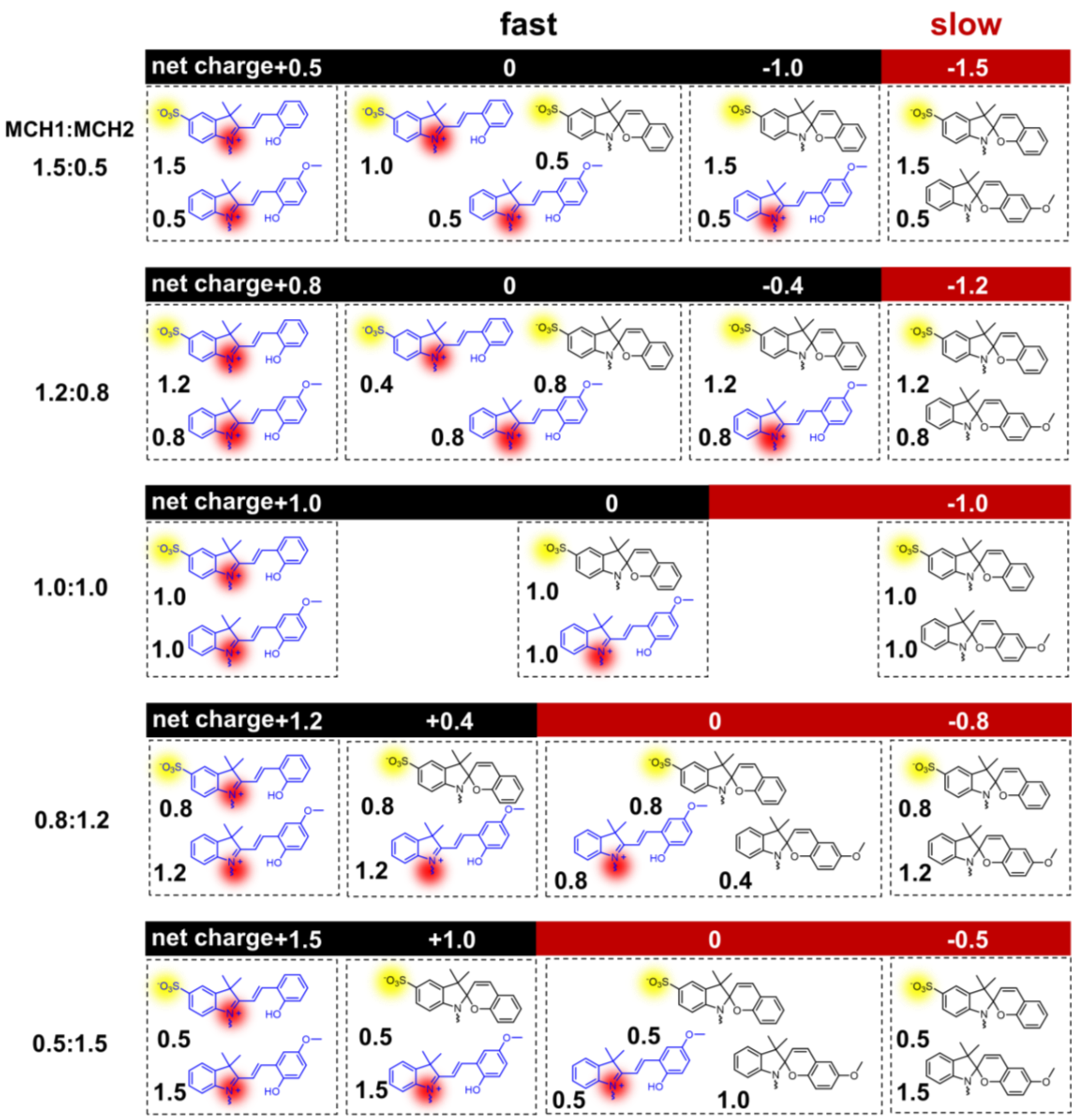

Supplementary Fig. 9. **Schematic representation of the photoisomerization process under light irradiation inside the MCH(1+2) hydrogel with variable mixing molar ratios, including 1.5:0.5, 1.2:0.8, 1.0:1.0, 0.8:1.2 and 0.5:1.5.** The net charge is listed on the top with the corresponding chemical structures and compositions shown below for each transient state. Black indicates a fast step, and red indicates a slow step.

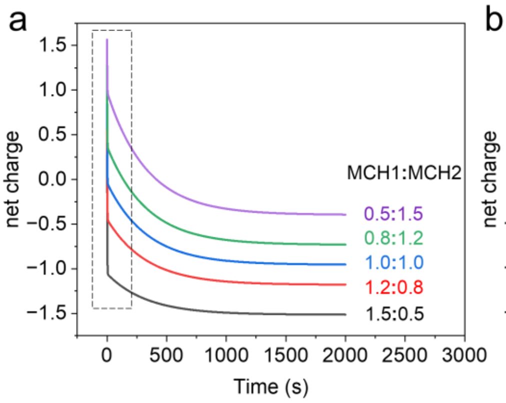

Supplementary Fig. 10. **Net charge calculation.** (a) Calculated net charge changes of MCH1 and MCH2 mixtures with variable mixing ratios as a function of irradiation time. (b) Zoomed-in area marked by a rectangular shape in (a). The simulation was performed using MATAB software.

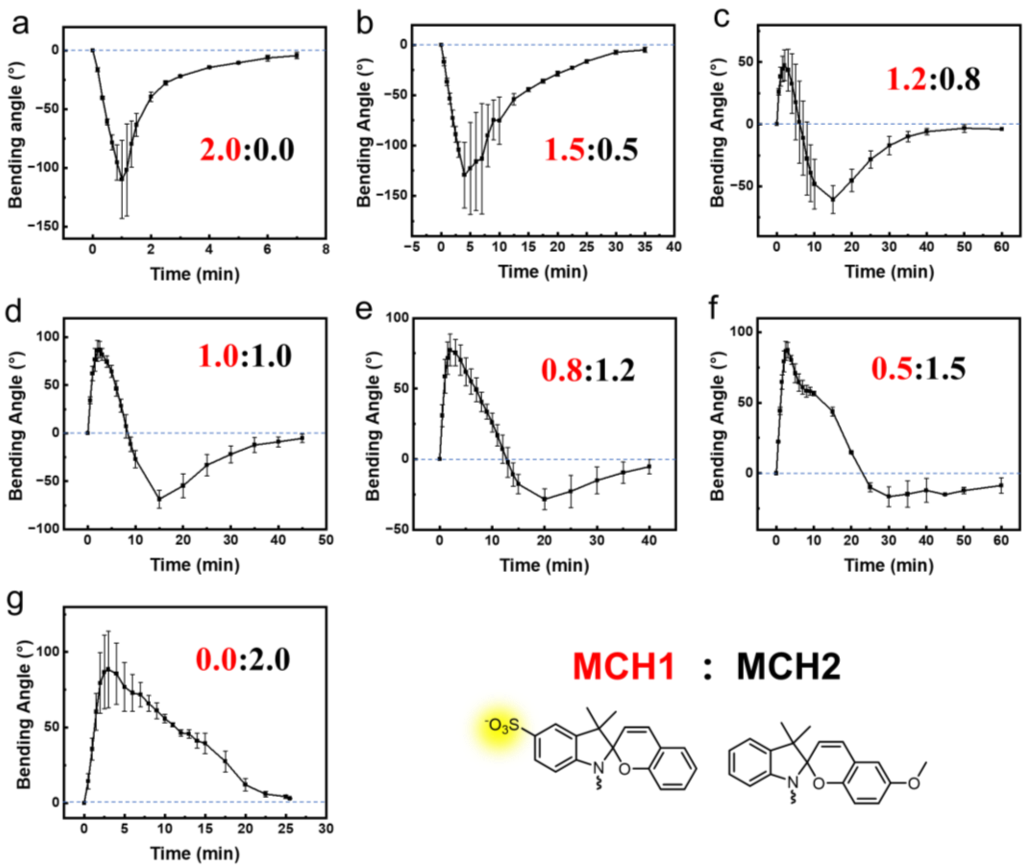

Supplementary Fig. 11. **Effect of mixing molar ratio on the bidirectional bending performance.** Plot of the transient bidirectional bending deformation progress of a ribbon-shaped MCH(1+2) hydrogel with variable mixing ratios upon irradiation from the left. (a) MCH1:MCH2 = 2.0:0.0, (b) MCH1:MCH2 = 1.5:0.5, (c) MCH1:MCH2 = 1.2:0.8, (d) MCH1:MCH2 = 1.0:1.0, (e) MCH1:MCH2 = 0.8:1.2, (f) MCH1:MCH2 = 0.5:1.5, (g) MCH1:MCH2 = 0.0:2.0. Error bars represent standard deviations of data collected from three separate samples.

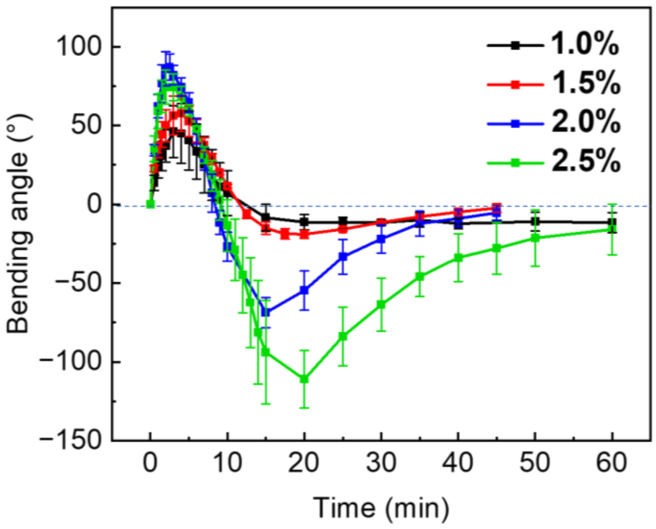

Supplementary Fig. 12. Plot of the bending angles of MCH(1+2) hydrogel ribbons with variable total grafting densities (MCH1:MCH2 = 1:1). Error bars represent standard deviations of data collected from three separate samples.

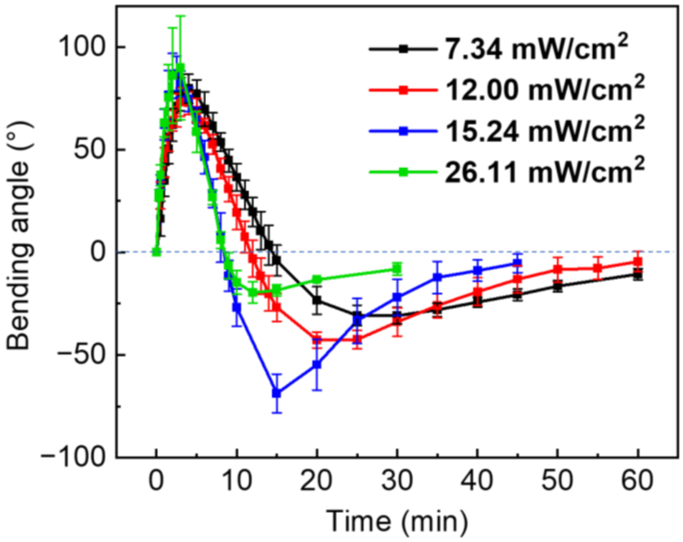

Supplementary Fig. 13. Plot of the bending angles of MCH(1+2) hydrogel ribbons containing a fixed mixing ratio of 1:1 and a fixed total grafting density of 2.0% upon irradiation with variable light intensities. Error bars represent standard deviations of data collected from three separate samples.

1'-(2-(methacryloyloxy)ethyl)-6-methoxy-3',3'-dimethylspiro[chromene-2,2' indoline]-5'-sulfonate **(MCH3)**

^{1}H-NMR (400 MHz, $CDCl_3$) δ(ppm): 7.70 (dt, J = 8.2, 1.9 Hz, 1H), 7.627.55 (m, 1H), 6.80 (d, J = 10.3 Hz, 1H), 6.67 (dt, J = 8.8, 2.5 Hz, 1H), 6.64-6.52 (m, 3H), 6.07 (d, J = 1.7 Hz, 1H), 5.70 (dd, J = 10.2, 2.0 Hz, 1H), 5.55 (q, J = 1.7 Hz, 1H), 4.34-4.20 (m, 2H), 3.75 (d, J = 2.0 Hz, 3H), 3.58 (dt, J = 15.3, 6.5 Hz, 1H), 3.40 (dt, J = 15.2, 6.2 Hz, 1H), 1.91 (t, J = 1.3 Hz, 3H), 1.28-1.25 (m, 3H), 1.12 (d, J = 2.1 Hz, 3H).

^{13}C-NMR (400 MHz, $CDCl_3$) δ(ppm): 153.30, 148.69, 147.90, 136.28, 136.11, 136.01, 129.55, 126.07, 125.88, 120.04, 119.96, 118.62, 115.50, 115.45, 111.57, 105.29, 104.35, 62.89, 55.78, 52.01, 42.34, 31.25, 25.59, 19.98, 18.37.

MS-ESI (m/z): [M] calc. for $C_{25}H_{26}NO_7S^-$ 484.54; $[M+3H]^{3+}$ found 487.60.

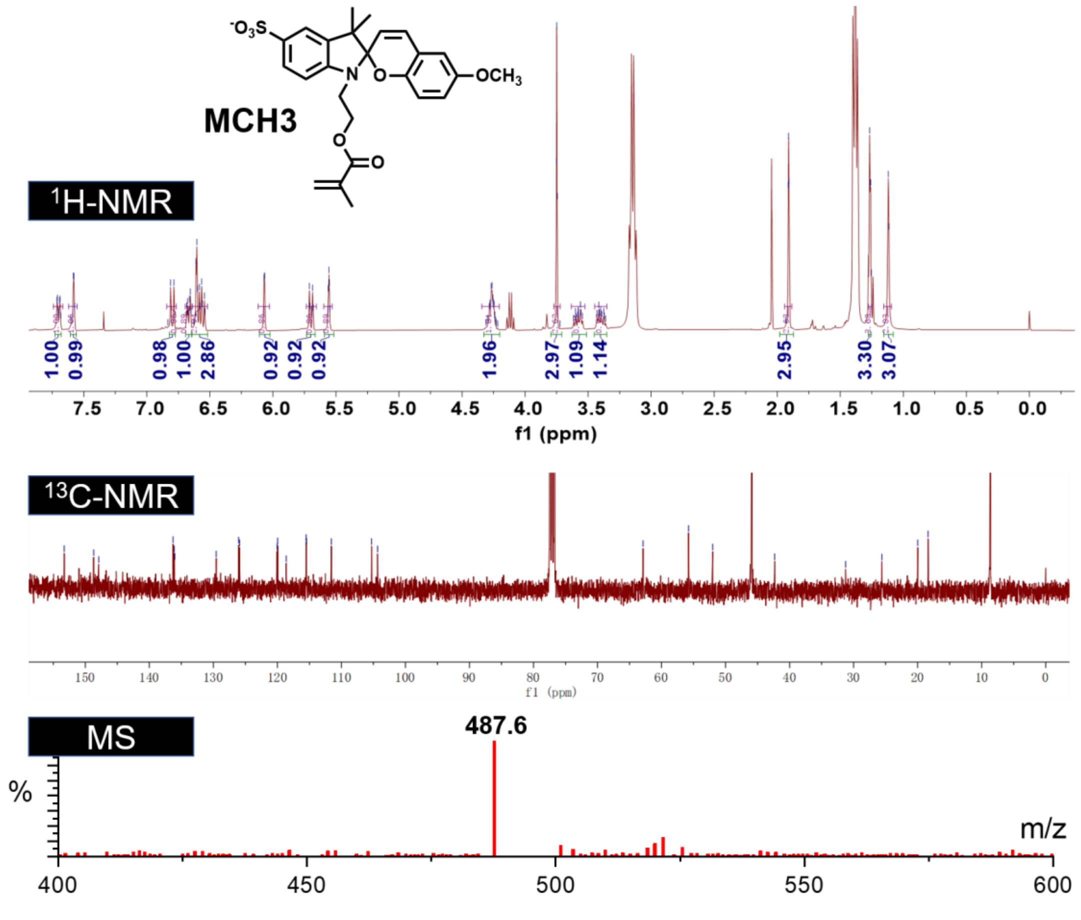

Supplementary Fig. 14. **Characterization of MCH3.** ^{1}H-NMR (top), ^{13}C NMR (middle) and MS spectra (bottom) of MCH3.

2-(3',3'-Dimethylspiro[chromene-2,2'-indolin]-1'-yl) ethyl methacrylate **(MCH4)**

^{1}H-NMR (400 MHz, $CDCl_3$) δ(ppm): 7.17 (td, J = 7.7, 1.3 Hz, 1H), 7.06 (dtd, J = 15.8, 7.7, 1.7 Hz, 3H), 6.88-6.78 (m, 3H), 6.72-6.63 (m, 2H), 6.08 (p, J = 1.1 Hz, 1H), 5.68 (d, J = 10.2 Hz, 1H), 5.55 (p, J = 1.6 Hz, 1H), 4.29 (t, J = 6.4 Hz, 2H), 3.60 (dt, J = 15.1, 6.7 Hz, 1H), 3.40 (dt, J = 15.1, 6.1 Hz, 1H), 1.92 (t, J = 1.3 Hz, 3H), 1.29 (s, 3H), 1.14 (s, 3H).

^{13}C-NMR (400 MHz, $CDCl_3$) δ(ppm): 167.33, 154.10, 147.23, 136.37, 136.18, 129.82, 129.47, 127.57, 126.79, 125.76, 121.74, 120.20, 119.53, 119.24, 118.47, 115.08, 106.46, 104.46, 63.02, 52.22, 42.42, 25.85, 20.08, 18.40.

MS-ESI (m/z): [M] calc. for $C_{24}H_{25}NO_3$ 375.47; $[M+H]^+$ found 376.19.

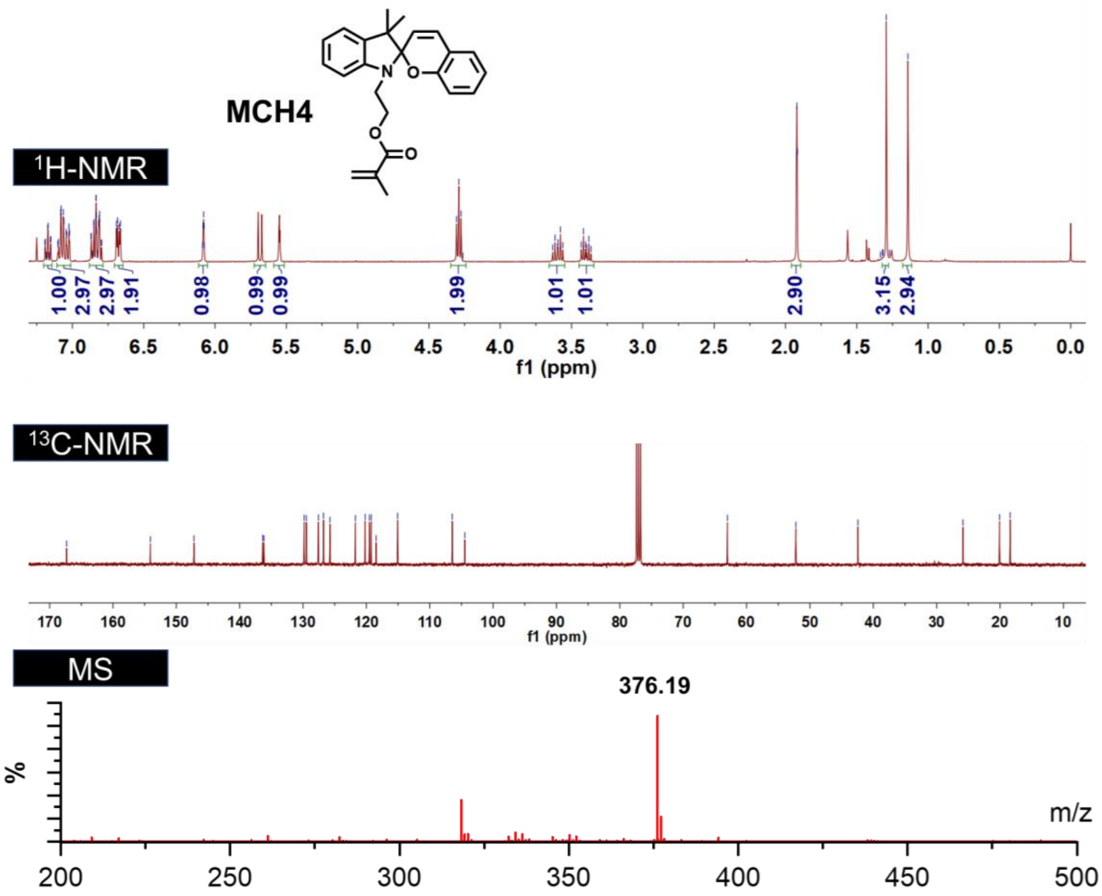

Supplementary Fig. 15. **Characterization of MCH4.** ^{1}H-NMR (top), ^{13}C NMR (middle) and MS spectra (bottom) of MCH4.

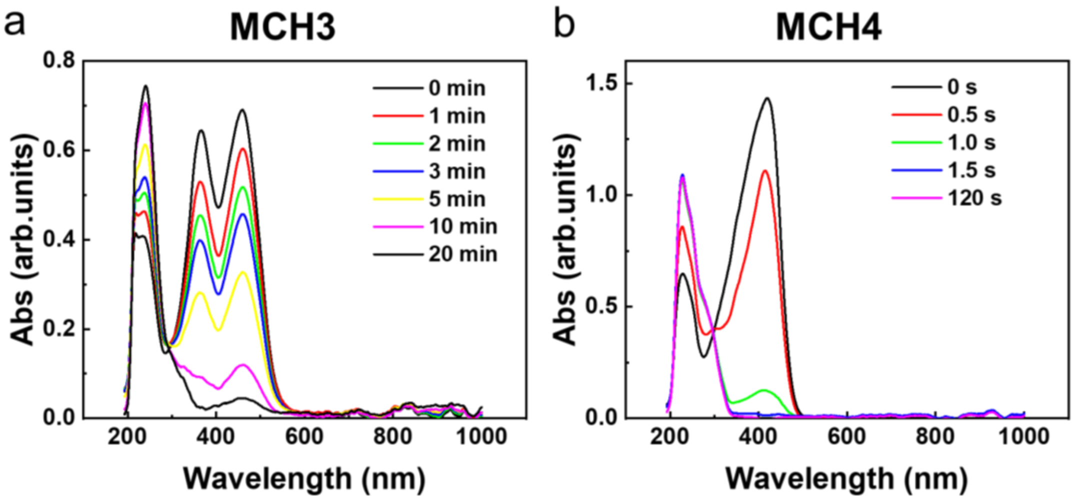

Supplementary Fig. 16. **UV–Vis absorption spectra.** (a) MCH3 and (b) MCH4 were measured in a solvent mixture of methanol/water (4:1, v/v) upon irradiation (450 nm, 154.6 mW/cm^2) for various durations.

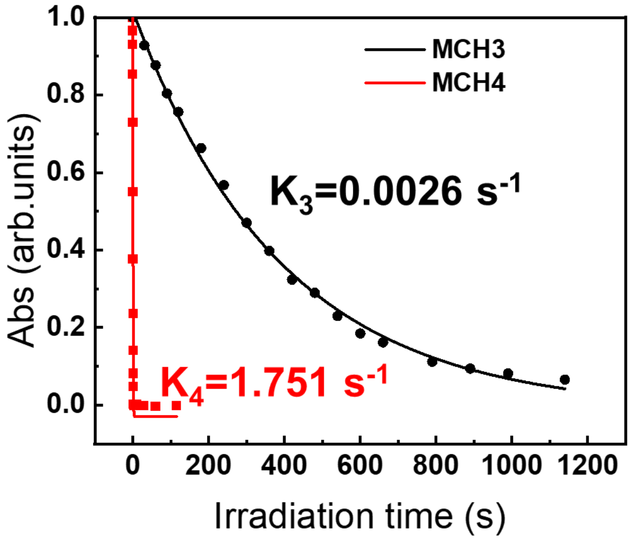

Supplementary Fig. 17. Photoisomerization kinetics study. Plot of the characteristic absorbances of MCH3 at 456 nm (red) and MCH4 at 422.5 nm (black) versus irradiation time (450 nm, 154.6 mW/cm^2), followed by fitting to the ExpDec1 function to obtain the photoisomerization rates.

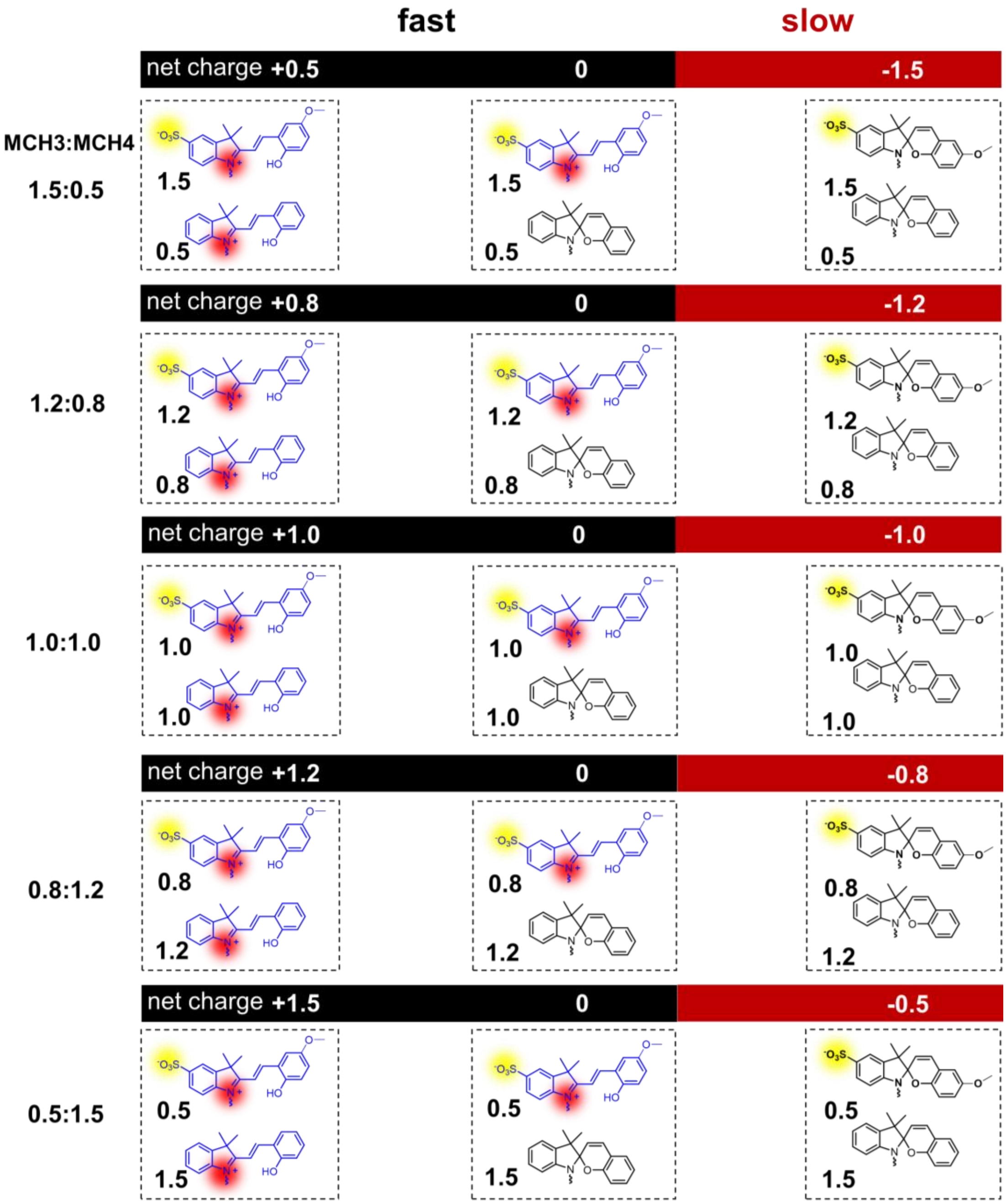

Supplementary Fig. 18. **Schematic representation of the photoisomerization process under light irradiation inside the MCH(3+4) hydrogel with variable mixing molar ratios, including 1.5:0.5, 1.2:0.8, 1.0:1.0, 0.8:1.2 and 0.5:1.5.** The net charge is listed on the top with the corresponding chemical structures and compositions shown below for each transient state. Black indicates a fast step, and red indicates a slow step.

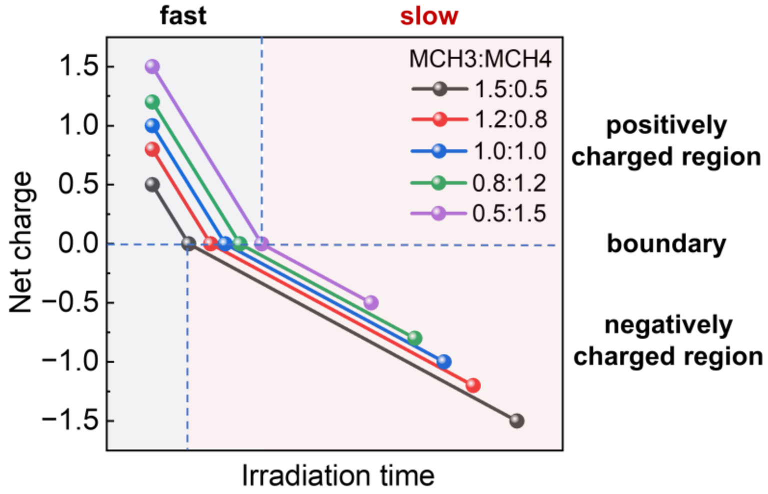

Supplementary Fig. 19. Plot of the net charge changes in mixed MCH3 and MCH4 with variable ratios as a function of irradiation time, showing positively and negatively charged regions with tunable charge transition time points.

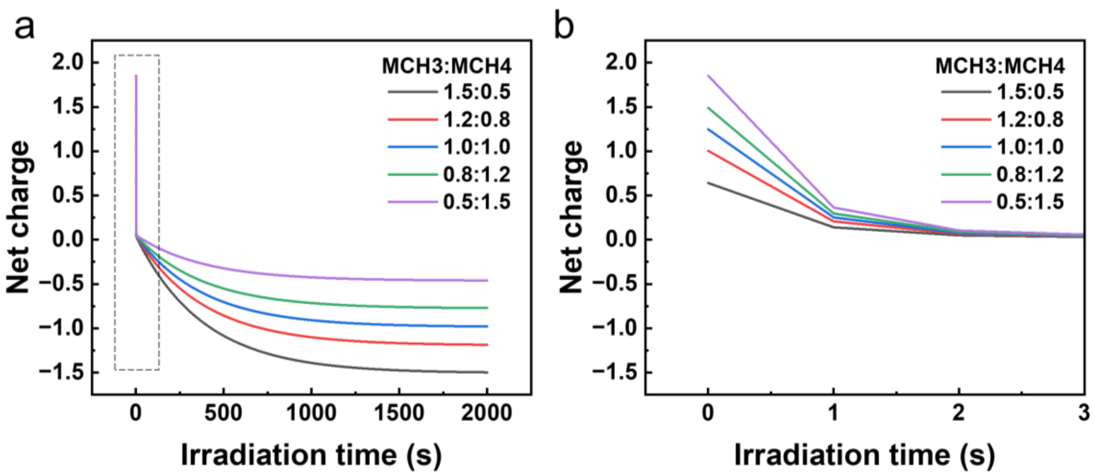

Supplementary Fig. 20. Net charge calculation. (a) Calculated net charge changes of MCH3 and MCH4 mixtures with variable mixing ratios as a function of irradiation time. (b) Zoomed-in area marked by a rectangular shape in (a). The simulation was performed using MATAB software.

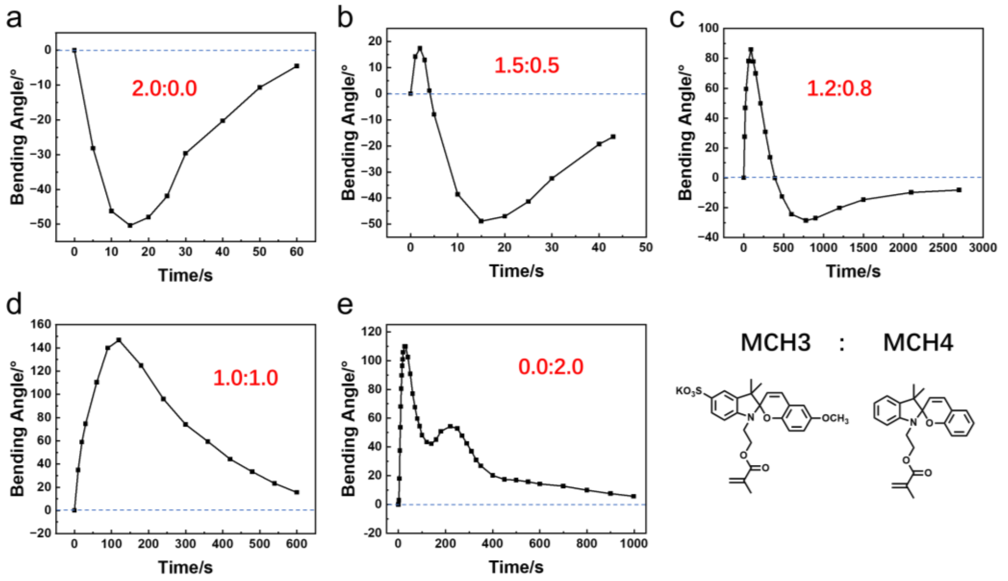

Supplementary Fig. 21. **Effect of mixing molar ratio on the bidirectional bending performance.** Plot of the transient bidirectional bending deformation progress of a ribbon-shaped MCH(3+4) hydrogel with variable mixing ratios upon irradiation from the left, (a) 2.0:0.0, (b) 1.5:0.5, (c) 1.2:0.8, (d) 1.0:1.0 and (e) 0.0:2.0.

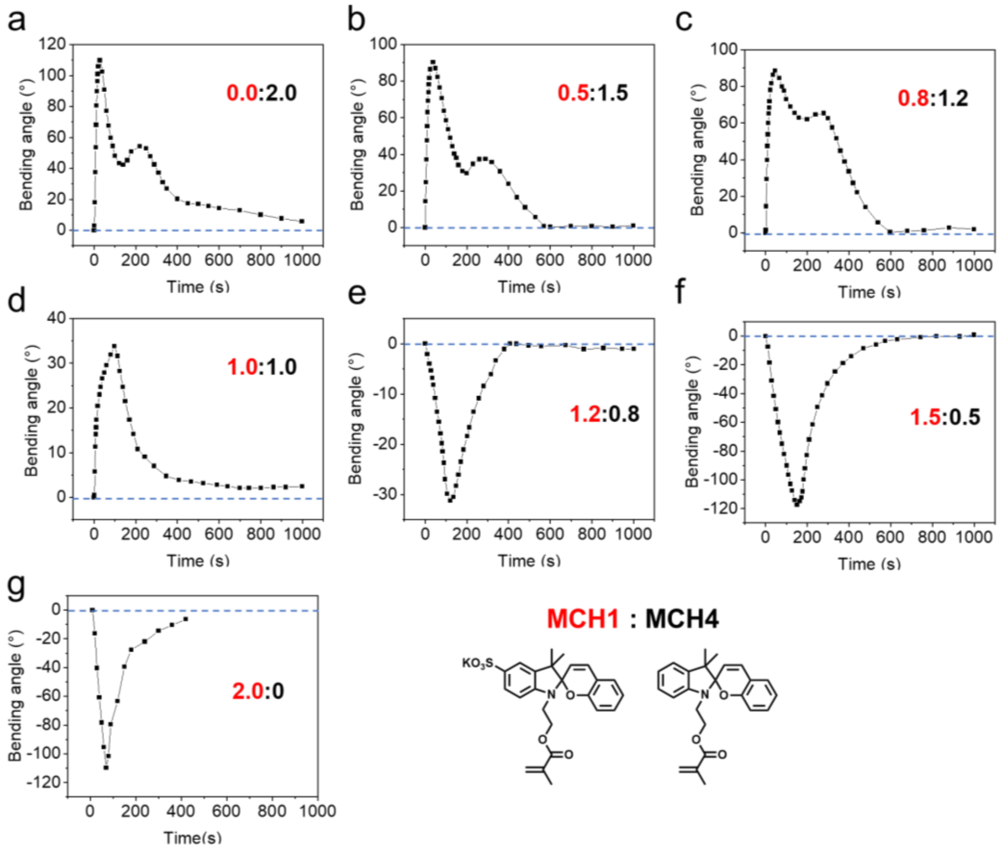

Supplementary Fig. 22. **Effect of mixing molar ratio on the bidirectional bending performance.** Plot of the bending deformation progress of a ribbon-shaped MCH(1+4) hydrogel with variable mixing ratios upon irradiation from the left, (a) 0.0:2.0, (b) 0.5:1.5, (c) 0.8:1.2, (d) 1.0:1.0, (e) 1.2:0.8, (f) 1.5:0.5 and (g) 2.0:0.0. No bidirectional bending was observed.

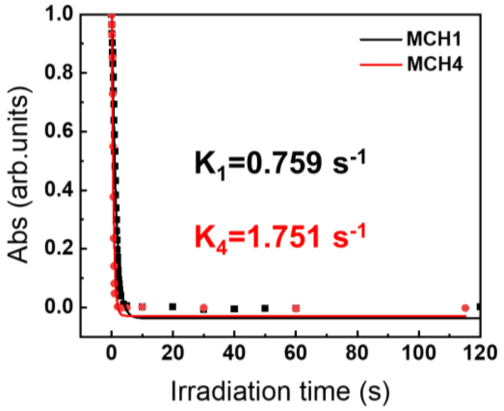

Supplementary Fig. 23. Photoisomerization kinetics study. Plot of the characteristic absorbances of MCH1 at 422.5 nm (black) and MCH4 at 422.5 nm (red) versus irradiation time (450 nm, 154.6 mW/cm^2), followed by fitting to the ExpDec1 function to obtain the photoisomerization rates.

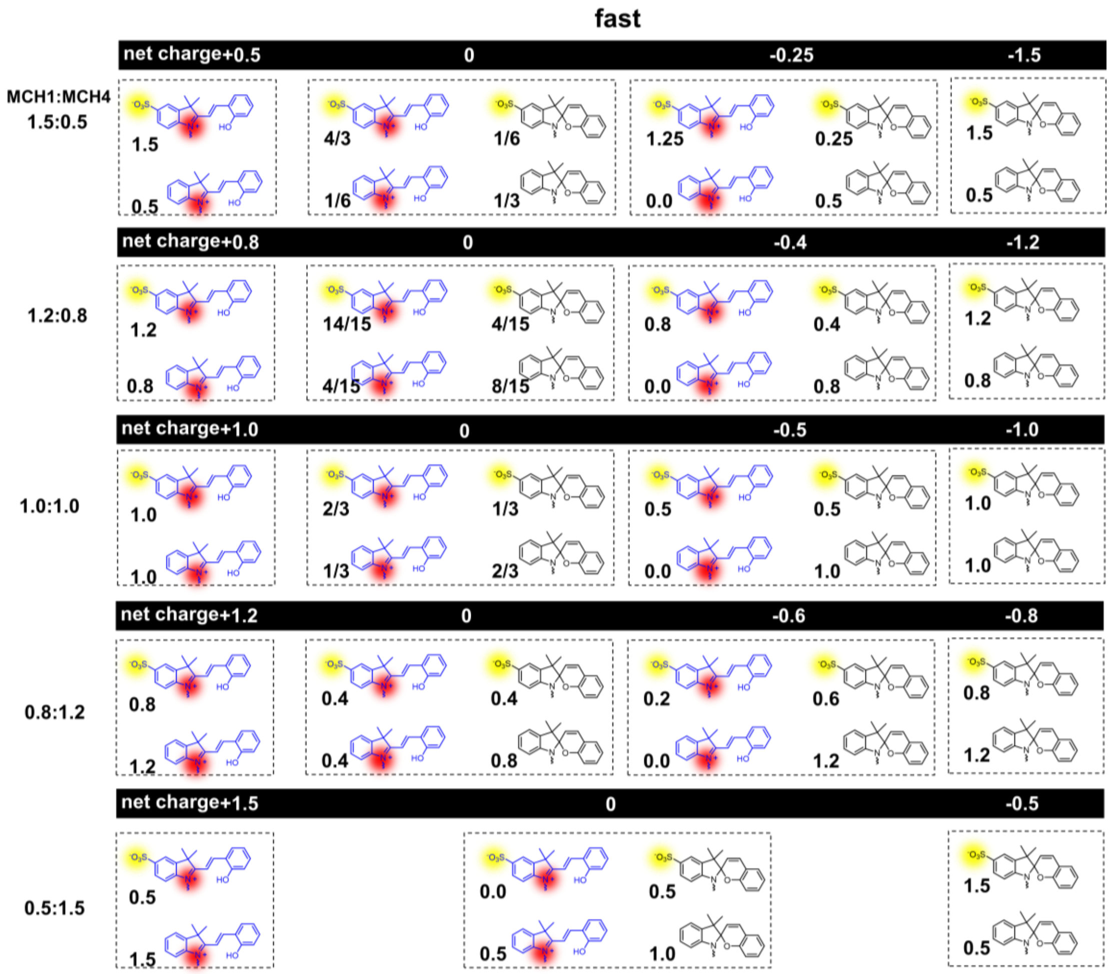

Supplementary Fig. 24. Schematic representation of the photoisomerization process under light irradiation inside the MCH(1+4) hydrogel with variable mixing molar ratios, including 1.5:0.5, 1.2:0.8, 1.0:1.0, 0.8:1.2 and 0.5:1.5. The net charge is listed on the top with the corresponding chemical structures and compositions shown below for each transient state. Black indicates a fast step.

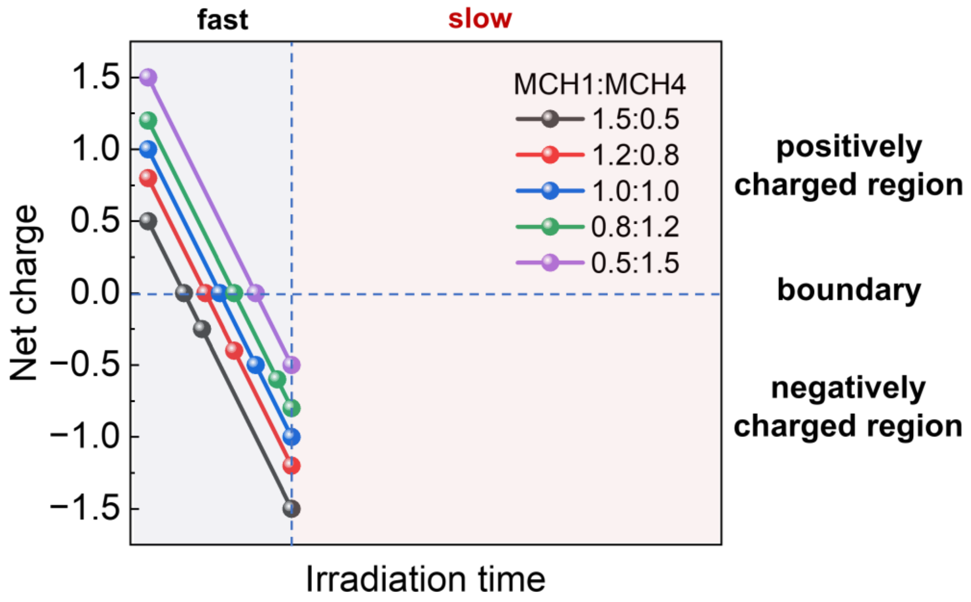

Supplementary Fig. 25. Plot of fast net charge changes in mixed MCH1 and MCH4 with variable ratios as a function of irradiation time, showing positively and negatively charged regions with tunable charge transition time points.

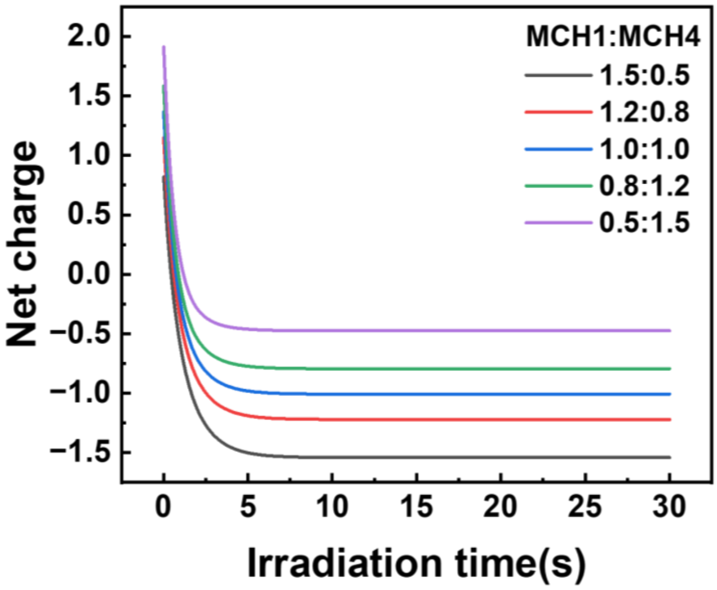

Supplementary Fig. 26. Calculated fast net charge changes of MCH1 and MCH4 mixtures with variable mixing ratios as a function of irradiation time.

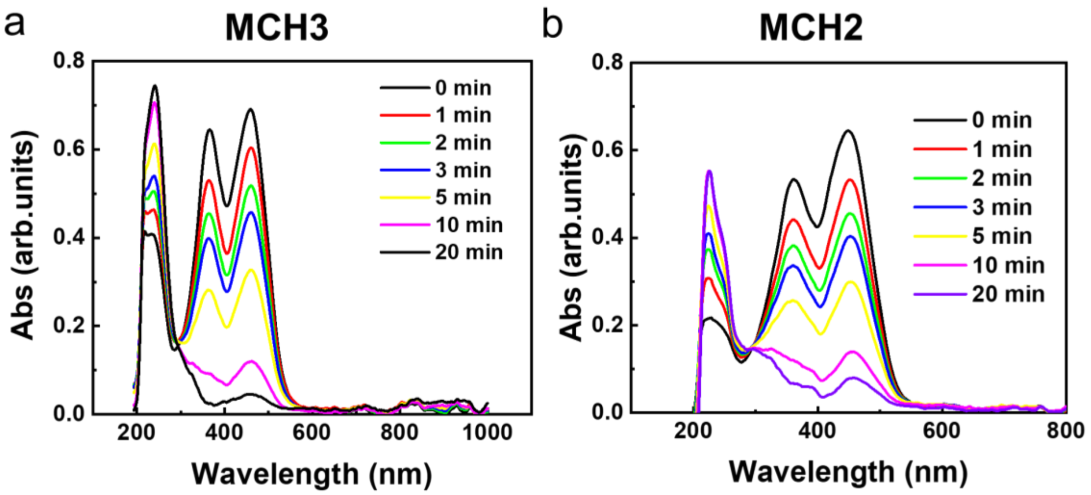

Supplementary Fig. 27. **UV–Vis absorption spectra.** (a) MCH3 and (b) MCH2 were measured in a solvent mixture of methanol/water (4:1, v/v) upon irradiation (450 nm, 154.6 mW/cm^2) for various durations.

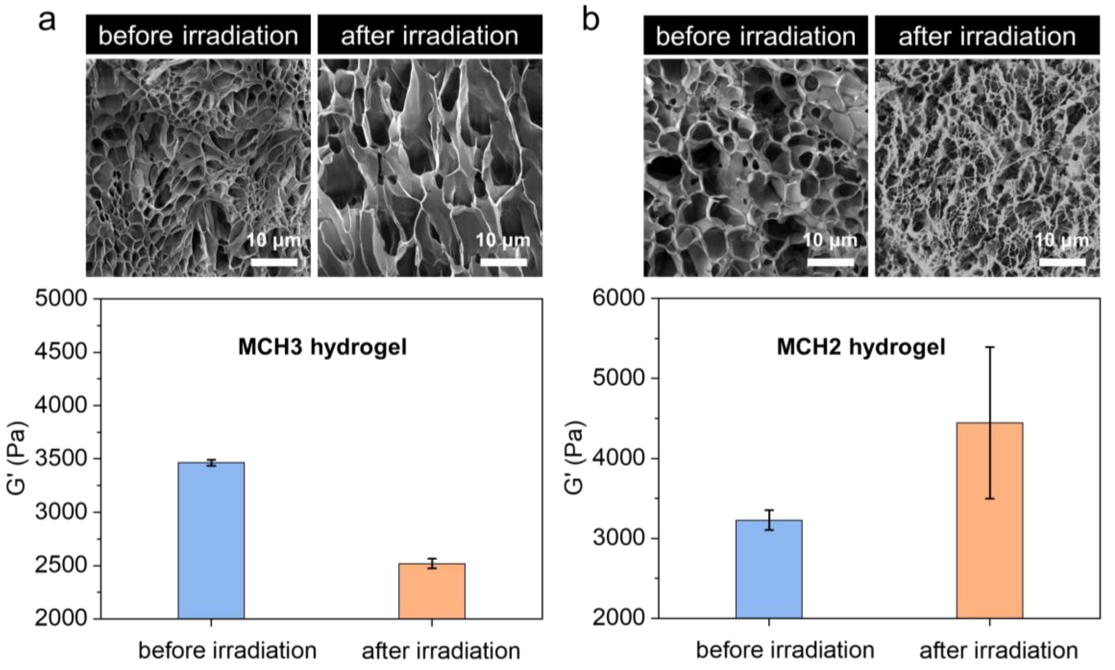

Supplementary Fig. 28. **Mechanical and morphological characterization of single-component hydrogel upon irradiation.** (a) Rheological measurements of single-component MCH3 hydrogels before (blue) and after irradiation (450 nm, 8.99 mW/cm²) for 90 min (orange). The top images are corresponding SEM images showing the changes in microporosity. (b) Rheological measurements of single-component MCH2 hydrogels before (blue) and after irradiation (450 nm, 8.99 mW/cm²) for 90 min (orange). The top images are corresponding SEM images showing the changes in microporosity. Error bars represent standard deviations of data collected from three separate samples.

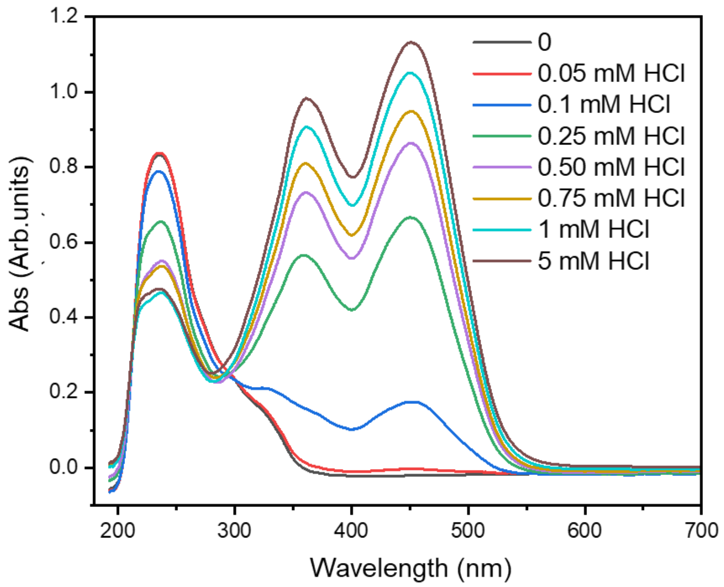

Supplementary Fig. 29. UV–Vis absorption spectra of mixed MCH3 and MCH2 with a fixed mixing molar ratio of 1:1 (total concentration of 0.1 mM) in a mixture of methanol/water (4:1, v/v) containing variable concentrations of HCl after equilibrium in the dark.

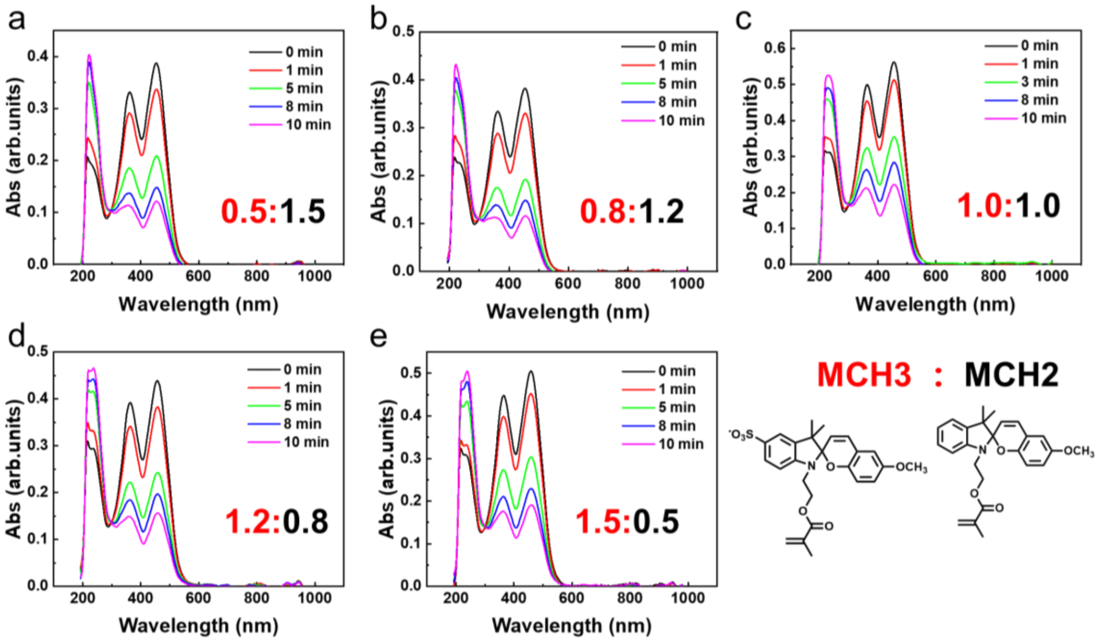

Supplementary Fig. 30. **UV–Vis absorption spectra of mixed MCH3 and MCH2 with variable mixing molar ratios, (a) 0.5:1.5, (b) 0.8:1.2, (c) 1.0:1.0, (d) 1.2:0.8 and (e) 1.5:0.5.** The measurements were performed in a solvent mixture of methanol/water (4:1, v/v) upon irradiation (450 nm, 154.6 mW/cm^2) for different durations.

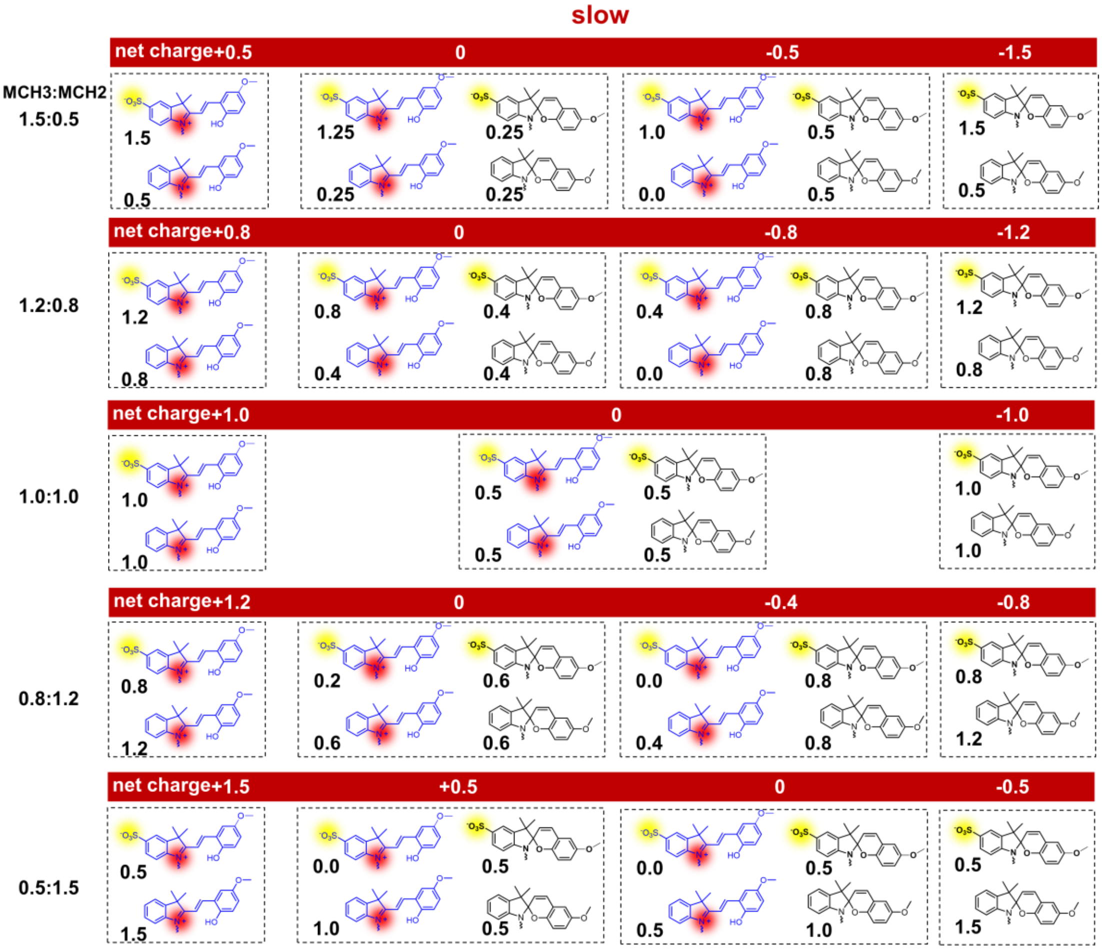

Supplementary Fig. 31. **Schematic representation of the photoisomerization process under light irradiation inside hydrogels containing mixed MCH3 and MCH2 at variable mixing molar ratios, including 1.5:0.5, 1.2:0.8, 1.0:1.0, 0.8:1.2 and 0.5:1.5.** The net charge is listed on the top with the corresponding chemical structures and compositions shown below for each transient state.

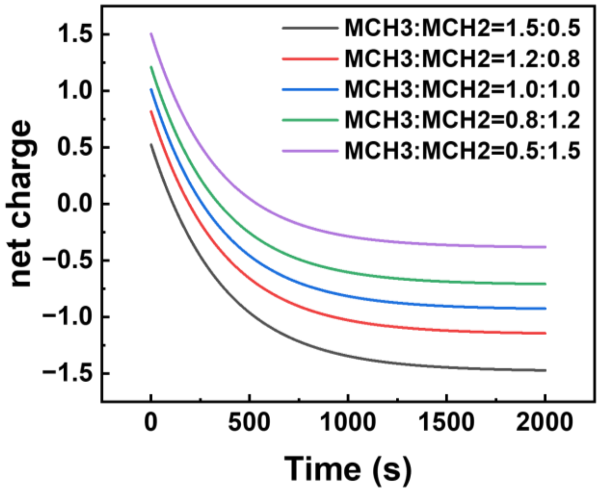

Supplementary Fig. 32. Calculated net charge changes of MCH3 and MCH2 mixtures with variable mixing ratios as a function of irradiation time.

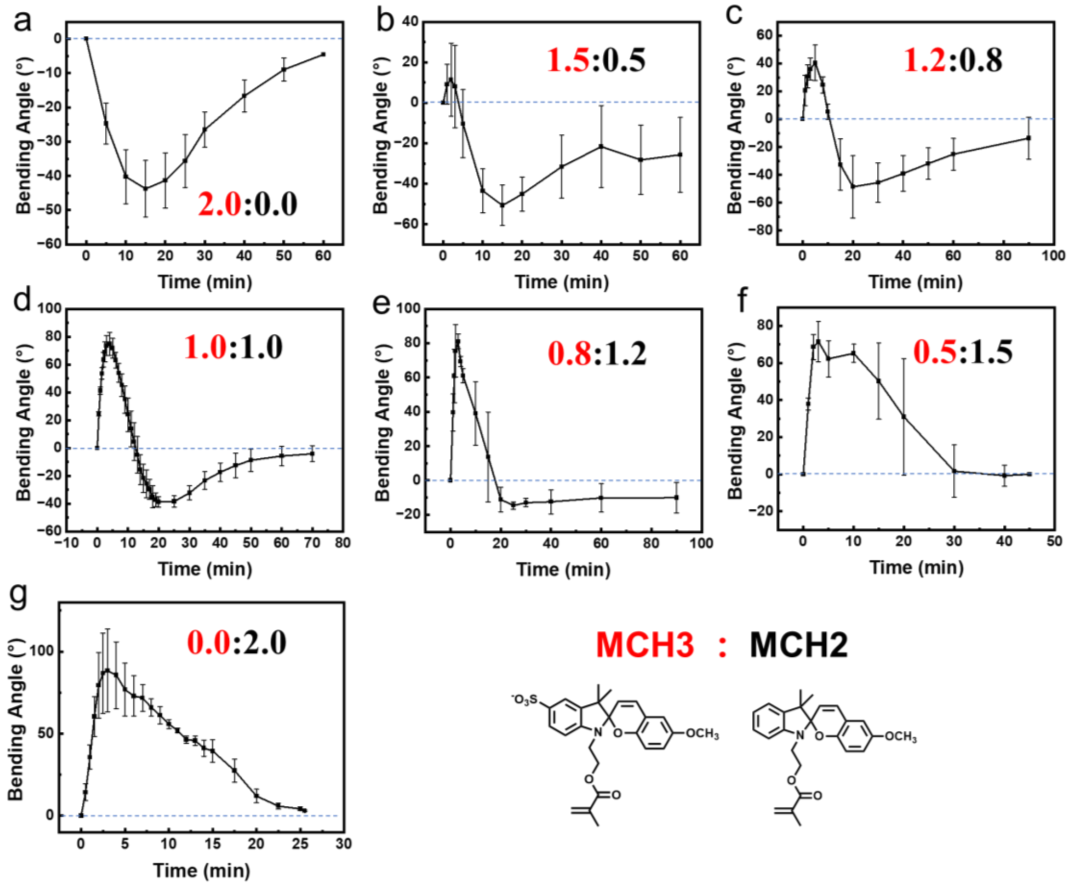

Supplementary Fig. 33. **Plot of the transient bidirectional bending deformation progress of a ribbon-shaped MCH(3+2) hydrogel with variable mixing ratios upon irradiation from the left.** (a) MCH3:MCH2 = 2.0:0.0, (b) MCH3:MCH2 = 1.5:0.5, (c) MCH3:MCH2 = 1.2:0.8, (d) MCH3:MCH2 = 1.0:1.0, (e) MCH3:MCH2 = 0.8:1.2, (f) MCH3:MCH2 = 0.5:1.5, (g) MCH3:MCH2 = 0.0:2.0. Error bars represent standard deviations of data collected from three separate samples.

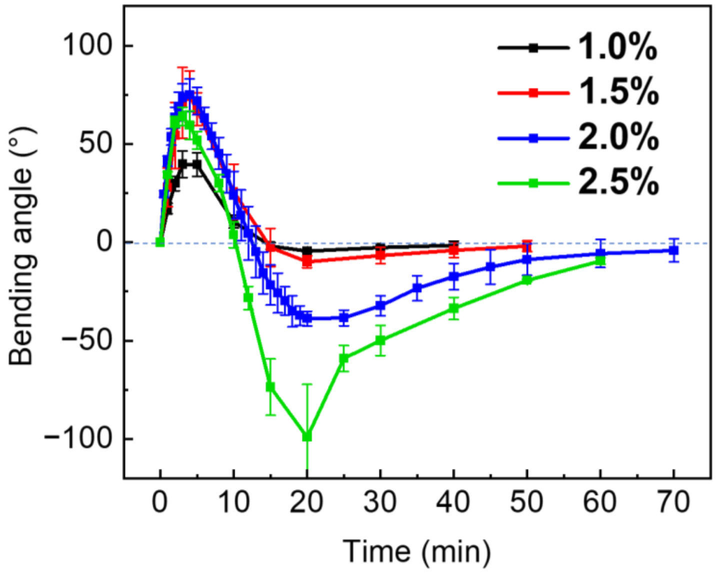

Supplementary Fig. 34. Plot of the bending angles of MCH(1+2) hydrogel ribbons with variable total grafting densities (MCH1:MCH2 = 1:1). Error bars represent standard deviations of data collected from three separate samples.

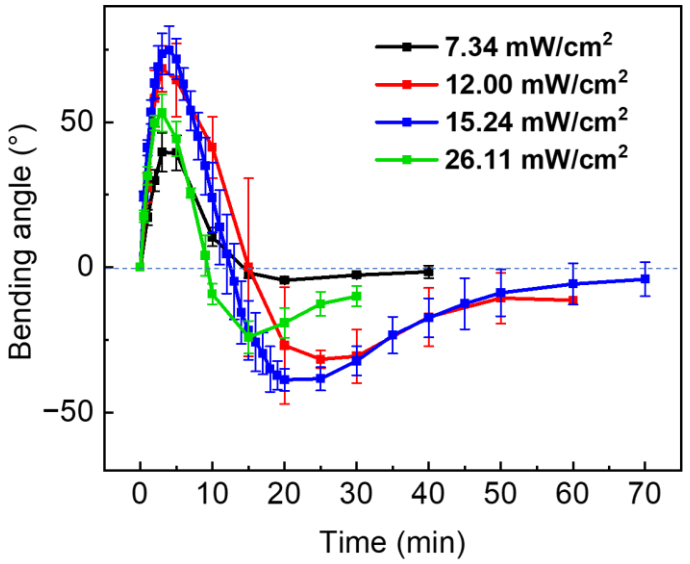

Supplementary Fig. 35. Plot of the bending angles of MCH(3+2) hydrogel ribbons containing a fixed mixing ratio of 1:1 and a fixed total grafting density of 2.0% upon irradiation with variable light intensities. Error bars represent standard deviations of data collected from three separate samples.

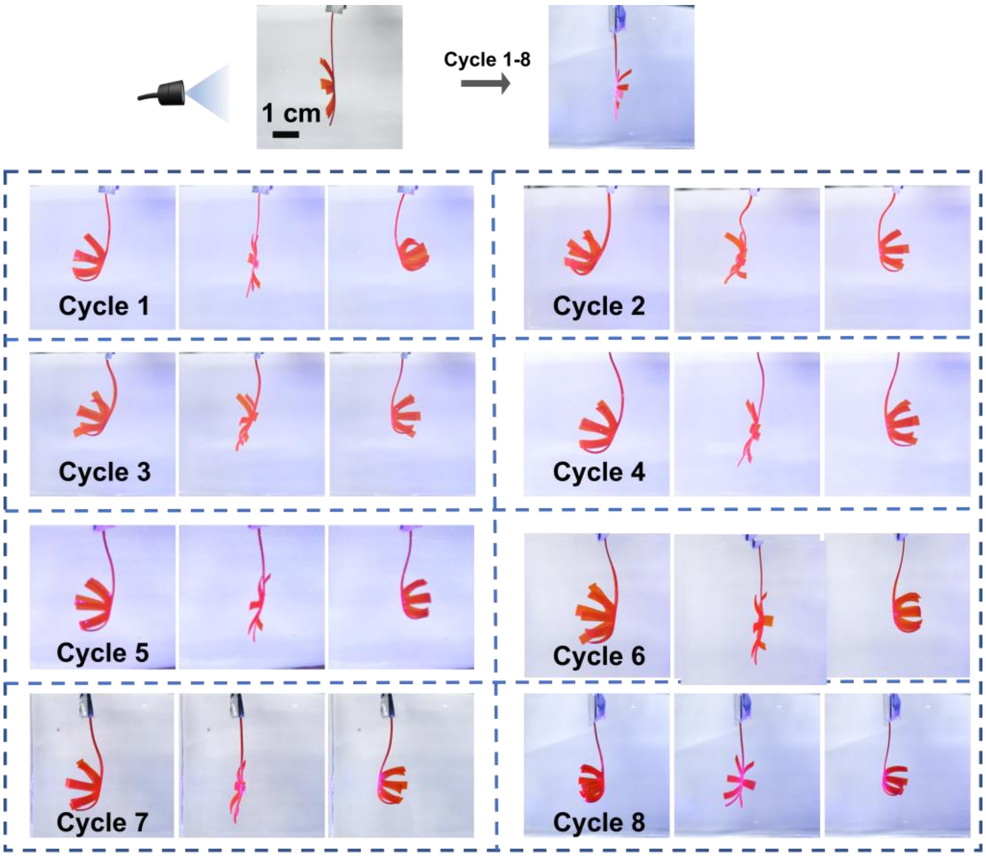

Supplementary Fig. 36. **Reversibility of bidirectional deformation.** Photographs of transient bidirectional positive and negative deformation cycles obtained using a star-shaped MCH(1+2) hydrogel with a fixed mixing ratio of 1.0:1.0 (MCH1:MCH2) and a total grafting ratio of 2.0% upon irradiation with constant irradiation (450 nm, 15.24 mW/cm^2).

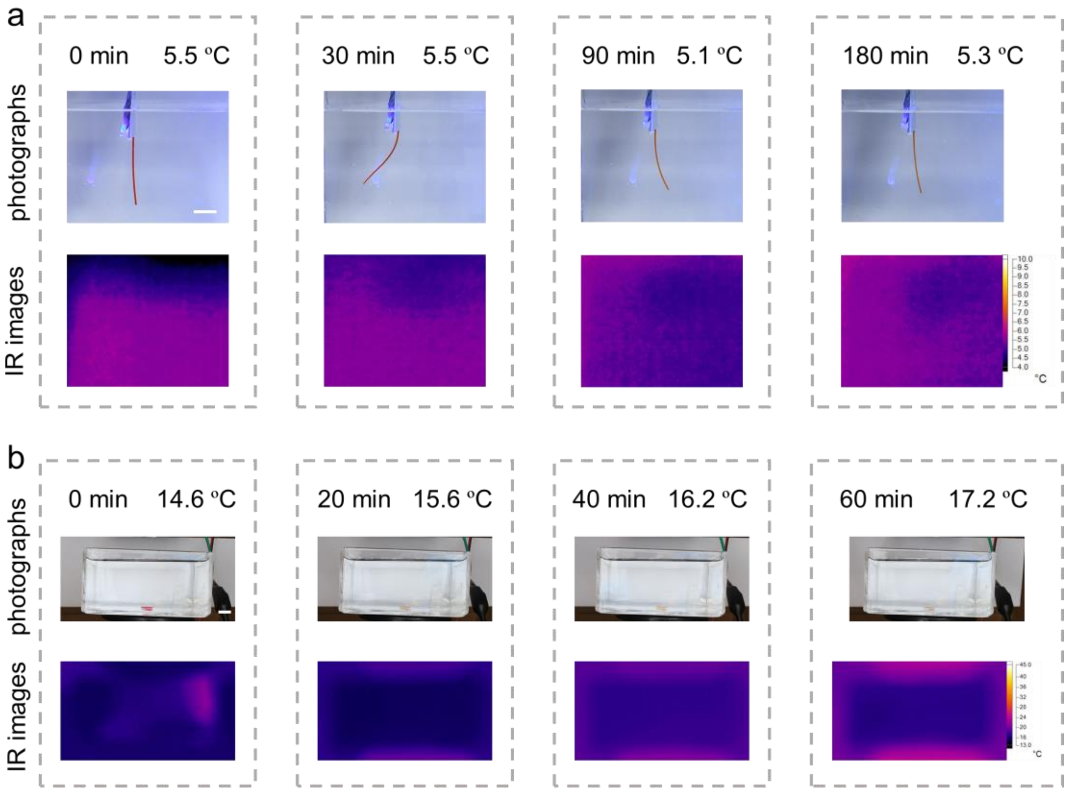

Supplementary Fig. 37. Exclusion of photothermal effect from the photoactuation process. (a) Measurements of the temperature change in the hydrogel ribbon after irradiation for different durations (450 nm, 15.24 mW/cm^2) in the photoactuation experiment of an MCH(1+2) hydrogel strip. (b) Changes in the temperature of the hydrogel dish with irradiation time (450 nm, 160.1 mW/cm^2) during preparation of the SEM sample using a dish MCH(1+2) hydrogel. Top, photographs of the hydrogel inside the water bath. Bottom, corresponding thermal image of the water bath with the average temperature shown on the top. The scale bar is 10 mm.
