# Supplementary material for: Self-regulated reversal deformation and locomotion of structurally homogenous hydrogels subjected to constant light illumination
Source: Nat Commun. 2024 Feb 24;15:1694. doi: 10.1038/s41467-024-46100-6 (PMC10894256; doi:10.1038/s41467-024-46100-6)
Supplement: Supplementary file 3 — Description of additional supplementary files [file 41467_2024_46100_MOESM3_ESM.pdf]

DESCRIPTION OF ADDITIONAL SUPPLEMENTARY FILES

Supplementary Movie 1

Self-regulated bidirectional bending deformation of hydrogel ribbons composed of two opposite spiroyrans selected from MCH1 to MCH4 upon irradiation with constant blue light from the left (450 nm, 15.24 mW/cm25). The total grafting ratio of the two spiropyrans was fixed at 2.0%.

Supplementary Movie 2

Self-regulated bidirectional deformation of MCH(1+2) hydrogels with different shapes upon irradiation with constant blue light from the left (450 nm, 15.24 mW/cm2 10). The mixing ratio of MCH1 to MCH2 was fixed at 1.0:1.0, and the total grafting ratio was 2.0%.

Supplementary Movie 3

Self-regulated nonmonotonous bidirectional rolling motion of an O-ring-shaped MCH(1+2) hydrogel under constant illumination from the left (450 nm, 10.67 mW/cm2).

Supplementary Movie 4

Monotonous unidirectional rolling toward the light source of an O-ring-shaped MCH2 hydrogel under constant illumination from the left (450 nm, 10.67 mW/cm2).

Supplementary Movie 5

Monotonous unidirectional rolling away from the light source of an O-ring-shaped MCH1 hydrogel under constant illumination from the left (450 nm, 10.67 mW/cm2).
